# The Impact of Diabetes Mellitus Duration and Complications on Health-Related Quality of Life Among Type 2 Diabetic Patients in Khamis Mushit City, Saudi Arabia

**DOI:** 10.7759/cureus.44216

**Published:** 2023-08-27

**Authors:** Jaber Abdullah Alshahrani, Ali Saad Alshahrani, Alaa Mohammed Alshahrani, Abdullah Mohammed Alshalaan, Maathir N Alhumam, Najim Z Alshahrani

**Affiliations:** 1 Family Medicine and Medical Education Department, Armed Forces Hospitals Southern Region, Khamis Mushit, SAU; 2 Preventive Medicine Department, Armed Forces Hospitals Southern Region, Khamis Mushit, SAU; 3 Family Medicine Department, Armed Forces Hospitals Southern Region, Khamis Mushit, SAU; 4 Endocrinology Department, Armed Forces Hospitals Southern Region, Khamis Mushit, SAU; 5 College of Medicine, King Faisal University, Alahsa, SAU; 6 Department of Family and Community Medicine, Faculty of Medicine, University of Jeddah, Jeddah, SAU

**Keywords:** quality of life improvement in diabetes, therapeutic lifestyle modifications, saudi arabia, abha, disease duration, autoimmune disease, type 2 dm, dm, diabetes, quality of life

## Abstract

Background and objectives: Diabetes mellitus is one of the most significant public health problems in Saudi Arabia. Therefore, this study aims to investigate the impact of disease duration and disease complications on health-related quality of life among type 2 diabetic patients.

Materials and methods: A cross-sectional study was conducted on 380 adult diabetic type 2 patients at a tertiary hospital in the city of Khamis Mushit in Saudi Arabia. The participants were asked to complete a pre-validated health status questionnaire (SF-36) consisting of 36 questions measuring eight domains of health, with each domain providing a score from 0 to 100. Demographic and clinical variables were collected using a diabetes type 2 specification form designed to be used in conjunction with the health status questionnaire. The clinical data included variables such as duration of diabetes, co-morbidities, and treatment modality. Statistical analysis was conducted using SPSS version 22 (IBM Corp., Armonk, NY, USA), with differences tested using various statistical tests. Spearman correlation was done between the score and continuous variables, such as age and BMI. A p-value less than 0.05 was considered statistically significant.

Results: Most of the participants (40%) were recently diagnosed with diabetes mellitus (less than one year ago) and 29.5% of the participants were diagnosed with diabetes mellitus within one to five years. The percentage of those with complications was 39.2%, which was mainly diabetic foot (43.4%) followed by nephropathy (29.5%). 46.8% of the participants were admitted due to conditions related to diabetes mellitus. Dietary modifications were prescribed in 38.4% of the participants, 19.5% used non-insulin medications only, 22.6% were on insulin, and 19.5% were using oral medications and insulin. The relationship between diabetes mellitus complications and quality of life domains revealed no significant difference in most of the domains except physical function and general health, which were lower with complicated diabetes melitus. Similarly, the relation between diabetes mellitus duration and quality of life domains was also not significant in all domains except physical function, which was low with a duration of more than 10 years.

Conclusions: Understandably, the complications associated with diabetes melitus resulted in low quality of life - in terms of physical function and general health - due to the organ-dysfunction associated with poor glycaemic control. Similarly, disease duration greater than 10 years resulted in impaired physical functioning.

## Introduction

Diabetes mellitus (DM) is a major cause of morbidity and mortality and is a great strain on public health funding [1]. The disease has reached epidemic proportions and more than 300 million people were affected by the year 2015 in the US alone [2]. The complications from diabetes have important effects on patients' quality of life, as well as socio-economic implications [3].

The Middle East is home to three of the world's top 10 diabetes-prevalence countries: the Kingdom of Saudi Arabia, Kuwait, and Qatar [2]. Specifically, diabetes rates in Saudi Arabia have increased almost 10-fold during the last three eras [3]. According to the International Diabetes Federation (IDF), one in every four Saudi adults will have diabetes by 2045 [4]. In Saudi Arabia, diabetes is rapidly reaching alarming proportions, becoming a major source of medical issues (such as heart attacks, kidney failure, stroke, etc.) and possibly death [4]. Moreover, DM creates a significant financial burden on individuals as well as healthcare systems [3,4]. In Saudi Arabia, the healthcare cost attributable to diabetes is projected to exceed $0.87 billion [4].

In response to the recognition of non-insulin dependent diabetes mellitus (NIDDM) as a major health problem in Saudi Arabia, Nozha et al. have proposed two goals for future research: (i) to reduce the number of new cases of NIDDM, especially through primary prevention, and (ii) to decrease the complications of the disease, with an overall goal of maximising the health status of the population [4]. Although improvements in the health status of diabetic patients may be assessed using typical clinical indicators, there is increasing awareness of the importance of measuring patient-reported outcomes, such as Quality of Life (QOL) [5]. Albeit there has been extensive analysis of the determinants, pathogenesis, and complications of diabetes in Saudi Arabia, we are unaware of any published assessments of health-related QOL (HRQoL) in this population. As QOL is an important predictor of an individual's ability to maintain long-term health, well-being, and productivity; improved QOL is the ultimate goal of all healthcare interventions, including diabetes management programs [6]. Given the huge health and economic burden of DM, understanding the QOL of patients with diabetes is important to help policymakers prioritize funding and implement interventions for the condition.

Diabetes and its management can have a considerable impact on people’s lives [7,8]; for example, it can lead to feelings of isolation, co-dependency, the experience of loss, overuse of defence mechanisms and loss of freedom, all of which can have consequences for the optimal management of the condition [5]. The literature on the impact of a range of interventions to improve care for people with diabetes has produced conflicting findings [9,10].

Haemoglobin A1c (HbA1c) levels, which reflect diabetic control, are inversely correlated with diabetic complications [11]. Directly quantifiable endpoints, such as microvascular disease, macrovascular disease and laboratory values, are typically assessed [12]. However, these do not reflect the patient's QOL. Furthermore, physician ratings of patients’ health do not correspond with patient ratings [13]. Since optimising QOL is a major goal of treatment, the relationship between traditional measures of diabetic control and patient-perceived QOL should be elucidated [14].

The relationship between disease duration, disease complication and QOL is unclear. Numerous studies have emphasised the significance of examining how type 2 diabetic patients in Saudi Arabia and other similar settings are affected by the length of the disease and its complications [15-18]. They both highlight the need for greater research on the subject and offer arguments in favour of its potential advantages for improving diabetes management and patient outcomes [19-21].

This study aims to investigate the impact of disease duration and disease complications on health-related quality of life among type 2 diabetic patients. The association of different socio-demographic characteristics with QOL in the Saudi population has also been studied.

## Materials and methods

A cross-sectional study was conducted among adult diabetic type 2 patients greater than 18 years of age attending the Diabetic Centre, Armed Forces Hospital, Southern Region-Khamis Hospital, KSA. The diabetes centre has 52 specialised clinics, including eight diabetes clinics for adults, six for paediatric diabetes, six for pregnant women, six for intensive insulin therapy, six for an insulin pump, and 10 clinics for diabetes complications for diseases (heart, kidneys, eyes, teeth), as well as 10 clinics for diabetic foot care, wounds, skin, nails, foot mechanics, gypsum and orthosis, and an oxygen therapy clinic. The sample size was calculated by Epi info software (version 7, CDC) by considering a 95% confidence interval and a 5% margin of error. Thus, the minimum sample of 380 diabetic patients was estimated. A systematic random sampling technique was adopted for a duration of 21 days to collect information from the estimated sample size. Eligible participants in this sample were asked to fill out the health status questionnaire on a single occasion. The self-administered pre-validated questionnaire consisted of several demographic and clinical variables. The core items from the SF-36.69 Arabic version of the questionnaire have been reported as valid and reliable [19]. The questionnaire itself consists of thirty-six questions measuring eight domains of health, namely, “physical functioning,” “role limitation due to physical health problems”, “bodily pain,” “general health,” “energy and vitality,” “social functioning,” “mental health,” and “role limitations due to mental health problems”. Each domain provides a score from 0 to 100, with zero indicating the worst health status and 100 the best. The percentage scores range from 0% (lowest or worst possible level of functioning) to 100% (highest or best possible level of functioning).

The study was conducted in accordance with the Declaration of Helsinki and approved by the Institutional Review Board of Armed Forces Hospitals Southern Region (Registration Number: H-06-KM-001).

Clinical and demographic variables were collected using the Diabetes Type 2 Specification form, which was developed by the researcher. The Diabetes Type 2 Specification form was designed to be used in conjunction with the SF-36 form [17]. Demographic variables such as gender, age, family income level, level of education, and the number and nature of chronic medical conditions were addressed. Clinical data were abstracted from the patient’s records, which included the following variables: duration of diabetes, co-morbidities, fasting or random capillary blood glucose measurements, HbAlc measurements in the previous six months, treatment modality (i.e., oral agents/insulin), and recent hospital admissions due to diabetes.

A pilot study on 40 patients was conducted to validate the questionnaire, to ensure that the questions were understandable. Ethical approval was obtained from the Research and Ethics Committee at Armed Forces Hospital, Southern Region-Khamis Hospital before the study commenced. Written informed consent was obtained from each participant to participate voluntarily in the study.

Statistical entry and analysis were performed using the Statistical Package for Social Science (version 22.0; IBM Corp., Armonk, NY, USA). The data presented as mean ± standard deviation for normally distributed variables and median (inter-quartile range) for abnormal distributions. Differences were tested using χ2 tests for categorical variables, Students’ t-tests (for normally distributed data) or Mann-Whitney tests (for not normally distributed data) for continuous variables in cases where two groups were compared, and ANOVA test (for normally distributed data) or the Kruskal-Wallis test (for not normally distributed data) for comparison of more than two groups. In the QOL domains, Spearman’s correlation was done between the score and continuous variables, such as age and BMI. A p-value less than 0.05 was considered statistically significant.

## Results

Three hundred eighty patients with type 2 diabetes participated in this study. Table 1 shows that the majority of the study’s participants were female (54.7%), had a university education (30.8%), were married (46.8%), had a job (63.7%), were between the ages of 20 and 30 (42.1%) and had an income of less than 5,000 Saudi riyal (SR; 36.8%); (100 USD = 375.04 SR as per Aug 21, 6:40 AM UTC). The table also shows that 29.8% of the study’s participants did not have any chronic diseases, while 27.4% had hypertension (HTN).

**Table 1:** Sociodemographic data of the study participants (n=380) SR: Saudi riyal, HTN: Hypertension, CVS: Cardiovascular

Sociodemographic variables		N	%
Gender	Male	172	45.3
	Female	208	54.7
Educational Status	Illiterate	30	7.9
	Primary	83	21.8
	Intermediate	27	7.1
	Secondary	83	21.8
	University	117	30.8
	Master's	40	10.5
Residency	Rural	73	19.2
	Urban	307	80.8
Marital status	Married	178	46.8
	Not Married	202	53.2
Job	Yes	242	63.7
	No	138	36.3
Age group	20-30	160	42.1
	31-40	29	7.6
	41-50	86	22.6
	51-60	69	18.2
	Above 60	36	9.5
Income	0-5,000 SR	140	36.8
	5,001-10,000 SR	121	31.8
	10,001-15,000 SR	85	22.4
	15,001-20,000 SR	31	8.2
	>20,000 SR	3	0.8
History of chronic diseases	No	112	29.8
	HTN	105	27.4
	CVS	39	10.4
	Renal	26	6.4
	Bronchial Asthma	34	8.5
	Allergy	18	4.8
	Others	48	12.8

The relationship between presence of DM complications and QOL domains revealed no significant difference in most of the domains, except physical function and general health, which were with presence of DM complications. Table 2 shows the DM history of the study’s participants. Most of the participants (40%) were recently diagnosed with DM (less than one year ago) and 29.5% of the participants were diagnosed with DM within one to five years. The percentage of those with complications was 39.2%, which was mainly diabetic foot (43.4%), followed by nephropathy (29.5%). 46.8% of the participants were admitted due to conditions related to DM.

**Table 2:** DM history of the study participants (n=380) DM: Diabetes mellitus, CVS: Cardiovascular

Variables		N	%
DM duration	Less than 1 year	152	40.0
	1-5 years	112	29.5
	5-10 years	81	21.3
	>10 years	35	9.2
DM complication	Yes	149	39.2
	No	231	60.8
Type of DM complication	Neuropathy	11	6.4
	Diabetic foot	75	43.4
	CVS complication	10	5.8
	Nephropathy	51	29.5
	Retinopathy	4	2.3
	Fatty liver	22	12.7
Admission	Yes	178	46.8
	No	202	53.2
Treatment	Diet	146	38.4
	Non-insulin medications	74	19.5
	Insulin	86	22.6
	Tablets + insulin	74	19.5
Following DM diet	Always	183	48.2
	Sometimes	146	38.4
	Never	51	13.4
Satisfaction about DM treatment	Totally satisfied	127	33.4
	Satisfied	126	33.2
	Neutral	56	14.7
	Dissatisfied	61	16.1
	Totally dissatisfied	10	2.6

Dietary modifications were prescribed in 38.4% of the participants, 19.5% used non-insulin medications only, 22.6% were on insulin, and 19.5% were using oral medications and insulin. The majority of the people (33.4%) were totally satisfied with their treatment. Another 33.2% were satisfied, 14.7% were neutral, 16.1% were dissatisfied, and 2.6% were totally dissatisfied.

The relation between DM duration (less than one year, one to five years, five to 10 years, and more than 10 years) and QOL domains among study participants is shown in Table 3. The results revealed that there was a statistically significant difference in QOL domains between the four groups, with the group with more than 10 years of DM having the lowest QOL scores.

**Table 3:** Relation between DM duration and QOL domains DM: Diabetes mellitus, QOL: Quality of life, F: One-way ANOVA, P: P-value * Statistically significant (p < 0.05).

DM duration	Less than year		1-5 years		5-10 years		More than 10 years		F	P
QOL	MEAN	SD	MEAN	SD	MEAN	SD	MEAN	SD		
Physical functioning	65.99	22.36	58.93	23.36	62.10	19.65	57.57	29.51	2.63	0.05*
Role limitations due to physical health	27.49	22.25	30.84	21.89	26.85	20.07	32.86	24.87	0.31	0.82
Role limitations due to emotional problem	31.14	22.68	31.25	22.25	35.80	26.18	31.43	25.69	0.22	0.88
Energy	51.97	12.93	55.54	15.86	52.41	14.36	56.00	20.28	1.70	0.17
Emotional well-being	61.89	12.23	62.18	14.37	60.94	12.81	60.69	14.79	0.22	0.89
Social functioning	57.48	20.00	57.25	20.18	57.41	21.08	60.36	25.27	0.22	0.88
Pain	72.47	21.94	70.11	24.10	72.96	19.53	81.57	19.77	2.44	0.06
General health	52.83	9.38	53.44	10.91	53.52	12.59	55.43	14.21	0.53	0.66

The relationship between presence of DM complications and QOL domains revealed no significant difference in most of the domains except physical function and general health, which were with presence of DM complications (Table 4).

**Table 4:** Relation between DM complication and QOL domains DM: Diabetes mellitus, QOL: Quality of life, t: Independent sample t-test, P: P-value * Statistically significant (p < 0.05).

DM complication	Yes		No		t	P
QOL	MEAN	SD	MEAN	SD		
Physical functioning	58.36	21.14	64.85	23.84	-2.71	0.007*
Role limitations due to physical health	31.91	22.68	26.86	20.56	1.16	0.247
Role limitations due to emotional problem	33.33	25.85	31.46	24.74	0.40	0.693
Energy	54.53	15.84	52.81	14.36	1.09	0.275
Emotional well-being	62.58	14.44	61.07	12.36	1.08	0.279
Social functioning	58.47	20.51	57.14	20.93	0.61	0.542
Pain	71.46	22.16	73.53	22.00	-0.89	0.373
General health	54.93	13.24	52.40	9.25	2.19	0.029*

## Discussion

The purpose of the current study was to investigate the connections between various diabetic complications, and the disease duration and QOL of diabetes patients. There were 380 diabetic participants in the study who were at least 18 years old. The study's findings show that most individuals had only recently been diagnosed with diabetes, and a sizable percentage of them also had co-morbidities, like diabetic foot and neuropathy. The sample cohort had poor glycaemic control, as seen by the high mean fasting blood sugar (FBS) levels and HbA1c.

A few studies on diabetics’ quality of life have revealed that a number of variables were linked to a lower quality of life in type 2 diabetes patients, including older age, female gender, lower education level, lower income, longer duration of diabetes, higher HbA1c levels, the presence of related complications, and co-morbidities like hypertension, depression, and anxiety. Higher education, income, regular physical activity, and social support are a few factors that have been linked to better quality of life in type 2 diabetes patients. Patients with type 2 diabetes may see an improvement in their quality of life as a result of interventions aimed at these controllable factors. When formulating patient-centred care plans for people with type 2 diabetes, healthcare providers should take these factors into account [22-27].

According to the study, low glycaemic control in diabetes patients is linked to lower QOL, as evidenced by the finding that QOL domains were adversely correlated with fasting blood glucose (FBG) and HbA1c levels. This result is in line with an earlier study that demonstrated a poor correlation between glycaemic control and QOL in diabetic individuals [28].

With the exception of social function, which was greater in men than women in terms of sociodemographic characteristics, the study found no discernible variation in QOL domains between genders. As with age groups and QOL domains, there was no statistically significant difference between them, but older groups, particularly those over 60, saw lower rates. These results are in line with an earlier study that found no appreciable distinction between male and female genders and age groups in QOL areas [29].

Additionally, the study discovered a substantial correlation between QOL domains and specific clinical variables. For instance, diabetic individuals with problems scored less well in the fields of physical function and general health. Similar to this, persons with diabetes for more than 10 years showed lower physical function scores. This result supports an earlier study that linked diabetic complications, having had diabetes for a longer time, and having QOL scores that were lower [30].

It is interesting to note that, with the exception of physical function, the study found that marital status was strongly associated with higher rates across all QOL domains. Urban residents showed significantly higher rates across all QOL domains as compared to non-urban residents. These results imply that social support and the home environment may be important in enhancing the QOL of diabetic patients [28-30].

With a mean EQ-5D-5L index score of 0.74, DM’s mild influence on HRQoL is indicated. Additionally, DM-related complication patients had significantly lower HRQoL than DM-free patients. The authors came to the conclusion that DM has a detrimental effect on HRQoL, particularly for patients with DM-related complications, and that primary healthcare providers should make prevention and management of DM-related problems a top priority in order to enhance the HRQoL of patients with DM [29-30].

Overall, the results of this study indicate that people with diabetes who have poor glycaemic control, complications, and a longer duration of their diabetes have lower QOL. The QOL of diabetic patients may also be improved by social support, a comfortable living environment, and treatment satisfaction. To enhance the QOL of diabetic patients, medical professionals should place a priority on enhancing glycaemic control and offering appropriate care for diabetes complications.

Interestingly, the effects of type 1 diabetes (T1D) with early onset and extended duration on German youths’ HRQoL have also been investigated. The authors discovered a decreased HRQoL, notably in the domains of physical functioning, emotional functioning, and treatment burden, was associated with early-onset and long-duration T1D. Additionally, compared to men and people with good metabolic control, women and people with poor metabolic control had significantly lower HRQoLs. The authors came to the conclusion that clinicians should place a high priority on the prevention and management of T1D-related complications, and that early and comprehensive diabetes management and psychosocial support are essential for improving the HRQoL of young people with T1D, particularly for those with early-onset and long-duration T1D [30].

However, it is important to note that this study has certain limitations. For example, the study was cross-sectional in design, which limits our ability to establish causal relationships between the factors examined and QOL. In addition, the study was conducted at a single centre, which limits the generalisability of the findings to other settings. Furthermore, the results should be interpreted with caution because we did not adjust the association for other confounding factors in patients. Future research should aim to overcome these limitations and explore further the associations between various factors and QOL in diabetic patients.

## Conclusions

This study investigated the relationship between duration and complication of DM to QOL among a diabetic population in Saudi Arabia. The findings of this study showed that the complications associated with DM resulted in low QOL in terms of physical function and general health, which was understandable due to organ dysfunctions associated with poor glycaemic control. Similarly, disease duration greater than 10 years resulted in impaired physical functioning. Further studies, that may incorporate additional factors like socio-demographics, physical activity, dietary habits, treatment satisfaction, etc., are warranted to better understand the factors of QOL among the diabetic population in Saudi Arabia.
